# lnc-MRGPRF-6:1 Promotes M1 Polarization of Macrophage and Inflammatory Response through the TLR4-MyD88-MAPK Pathway

**DOI:** 10.1155/2022/6979117

**Published:** 2022-01-28

**Authors:** Dan Hu, Yuzhong Wang, Zhihuan You, Yingfei Lu, Caihong Liang

**Affiliations:** ^1^Department of Cardiology, The Affiliated Jiangning Hospital of Nanjing Medical University, Nanjing 210000, China; ^2^Department of Geriatrics, The Affiliated Jiangning Hospital of Nanjing Medical University, Nanjing 210000, China; ^3^Central Laboratory, Translational Medicine Research Center, The Affiliated Jiangning Hospital of Nanjing Medical University, Nanjing 210000, China

## Abstract

**Background:**

Macrophage-mediated inflammation plays an essential role in the development of atherosclerosis (AS). Long noncoding RNAs (lncRNAs), as crucial regulators, participate in this process. We identified that lnc-MRGPRF-6:1 was significantly upregulated in the plasma exosomes of coronary atherosclerotic disease (CAD) patients in a preliminary work. In the present study, we aim to assess the role of lnc-MRGPRF-6:1 in macrophage-mediated inflammatory process of AS.

**Methods:**

The correlation between lnc-MRGPRF-6:1 and inflammatory factors was estimated firstly in plasma exosomes of CAD patients. Subsequently, we established lnc-MRGPRF-6:1 knockout macrophage model via the CRISPR/Cas9 system. We then investigated the regulatory effects of lnc-MRGPRF-6:1 on macrophage polarization and foam cell formation. Eventually, transcriptome analysis by RNA sequencing was carried out to explore the contribution of differential genes and signaling pathways in this process.

**Results:**

lnc-MRGPRF-6:1 was highly expressed in the plasma exosomes of CAD patients and was positively correlated with the expression of inflammatory cytokines in plasma. lnc-MRGPRF-6:1 inhibition significantly reduced the formation of foam cells. The expression of lnc-MRGPRF-6:1 was upregulated in M1 macrophage, and lnc-MRGPRF-6:1 knockout decreased the polarization of M1 macrophage. lnc-MRGPRF-6:1 regulates macrophage polarization via the TLR4-MyD88-MAPK signaling pathway.

**Conclusions:**

lnc-MRGPRF-6:1 knockdown can inhibit M1 polarization of macrophage and inflammatory response through the TLR4-MyD88-MAPK signaling pathway. lnc-MRGPRF-6:1 is a vital regulator in macrophage-mediated inflammatory process of AS.

## 1. Introduction

Coronary atherosclerotic disease (CAD) is a serious threat to human health, which is also one of the leading causes of human death worldwide [1]. Atherosclerosis (AS) is the pathological basis of CAD [2]. AS is a chronic inflammatory vascular disease, and macrophage plays a key role in the occurrence and development of AS. Apoptosis of macrophage is the process of forming necrotic core, and it is an inducer of long-term low-level inflammation of the intima, which can further promote the occurrence of AS [3]. Macrophage can secrete a variety of proinflammatory factors and chemokines to regulate the development of AS. After lipopolysaccharide (LPS) stimulation, macrophages initiate a series of inflammatory responses by activating a specific signaling cascade and releasing preinflammatory cytokines and mediators, including interleukin- (IL-) 6, tumor necrosis factor- (TNF-) *α*, reactive oxygen species (ROS), nitric oxide (NO), and prostaglandin E2 (PGE2) [4].

Long noncoding RNAs (lncRNAs) are a class of noncoding RNAs with a length of more than 200 nucleotides, which have important functions in many biological processes [5]. Previous studies show that lncRNAs are specifically expressed in various diseases, such as cancer, diabetes, cardiovascular disease, lung disease, and tissue fibrosis [6–8]. It is demonstrated that lncRNAs play important regulatory roles in inflammatory diseases by regulating macrophage function. It was reported that IL-7-AS promotes the expression of several inflammatory genes, including CCL2, CCL5, CCL7, and IL-6, by regulating NF-*κ*B and MAPK signaling pathways in macrophages [9]. FOXP1-IT1 overexpression attenuates THP-1 cell differentiation and inhibits inflammatory development [10]. Therefore, lncRNA is an important regulator of macrophage activation. In our preliminary work, we identified that lnc-MRGPRF-6:1 was highly expressed in plasma exosomes of CAD patients. Interestingly, further research demonstrated lnc-MRGPRF-6:1 was positively correlated with TNF-*α*, TNF-*β*, and CXCL11 in CAD patients, which indicates that lnc-MRGPRF-6:1 may participate in the regulation of macrophage-mediated inflammation in AS.

Dysregulation of phenotypic transitions in macrophages prolongs inflammation and impedes tissue repair. There is evidence that the polarization imbalance of M1/M2 macrophage may contribute to inflammatory diseases, including AS [11]. The polarization of M1 and M2 macrophages largely determines the development direction of AS inflammation [12]. During the development of AS, M1 and M2 macrophages show different position preferences on the vascular wall. The plaque shoulder is dominated by atherogenic M1 cells, which are relatively fragile and prone to rupture but are rarely found in the fibrous cap area. The harmful effects of M1 macrophage were offset by the recovery of M2 macrophage and the protective effects of AS [13]. The plasticity of these subpopulations may have a considerable influence on the outcome of AS. Overactivation of M1 may preferentially promote persistent inflammation and plaque progression.

Coincidentally, several researches have indicated that lncRNAs are involved in the dysregulation of phenotypic transitions in macrophages. Cao et al. demonstrated that lncRNA-MM2P could affect macrophage M2 polarization by regulating the dephosphorylation of signal transducer and activator of transcription 6 (STAT6) [14]. Chi et al. suggested that lncRNA GAS5 could act as a ceRNA to facilitate SOCS3 expression by sponging miR-455-5p and result in macrophage M2 polarization in childhood pneumonia [15]. Li et al. proved that lnc-BAZ2B promoted M2 macrophage activation primarily by upregulating the transcription of interferon regulatory factor 4 (IRF4), a key transcription factor for M2 macrophage activation [16].

Taken together, the regulation of macrophage polarization largely determines macrophage-mediated inflammation development and AS progression, and maybe some lncRNAs are the keys to the lock of macrophage polarization. Therefore, in the present study, we aim to explore the role of lnc-MRGPRF-6:1 in macrophage polarization and macrophage-mediated inflammation.

## 2. Materials and Methods

### 2.1. Cell Lines and Cell Culture

THP-1 cells (purchased from Shanghai Institute of Cell Research, Chinese Academy of Sciences) were incubated in RPMI Medium 1640 (Invitrogen, 11875-093) with 10% fetal bovine serum (Invitrogen, 21985). All cells were cultured in a moist environment at 37°C and 5% CO_2_.

### 2.2. Study Population

This study enrolled 20 CAD patients in the Cardiology Department of the Affiliated Jiangning Hospital of Nanjing Medical University. Inclusion criteria were patients undergoing coronary angiography for chest pain or suspected CAD. Coronary angiography of the patient showed a lesion stenosis ≥ 50% in any coronary artery. And patients with congenital heart disease, cardiomyopathy, liver and kidney insufficiency, blood system diseases, malignant tumors, and other concomitant diseases were excluded. 20 healthy controls were selected from physical examination people during the same period. Plasma samples were collected and stored at liquid nitrogen immediately after collecting and centrifugation.

### 2.3. Exosome Isolation

Plasma exosomes were isolated using an exosome extraction kit (Invitrogen, 4484450). The enrichment of exosomes was identified using transmission electron microscopy (TEM).

### 2.4. Isolation of Human Monocyte

Human monocyte was isolated from venous blood by Ficoll (Solarbio, p8900). CD14 microbeads (Invitrogen, 11367D) and magnetic beads conjugated with antibodies against human CD14 were used for the monocyte separation.

### 2.5. Establishment of Macrophage Polarization and Repolarization Models

THP-1 cells (2 × 10^5^/well) were inoculated in 24-well plates with 1 mL per well and incubated with PMA (Beyotime, S1819) with a final mass concentration of 100 ng/mL for 48 h. Human monocyte was incubated with macrophage colony-stimulating factor (M-CSF) (Beyotime, P5313) with a final mass concentration of 50 ng/mL for 7 days. M1 polarization of THP-1-derived macrophage and human monocyte-derived macrophage was performed using 20 ng/mL recombinant human IFN-*γ* (Beyotime, P5664) and 100 ng/mL LPS (Invitrogen, 00-4976). M2 polarization was performed using 20 ng/mL recombinant IL-4 (Beyotime, P5129). Nonpolarized PMA-activated cells were used as control. The polarization model of macrophage was established after 20 h of polarization. Based on the establishment of the macrophage polarization model, M1 macrophage was stimulated with 20 ng/ml IL-4 for 20 h and then induced to M2 polarization. M2 macrophage was induced to polarize toward M1 after 20 ng/mL IFN-*γ* and 100 ng/mL LPS stimulation for 20 h.

### 2.6. Establishment of Macrophage Foam Cell Model

As mentioned above, macrophage was activated from THP-1 cells by incubating with PMA. Macrophage-derived foam cell model was established by inducing 50 mg/L ox-LDL (Invitrogen, 2188176) for 24 h.

### 2.7. Generation and Infection of Lentivirus

CRISPR/Cas9 system was used to generate lnc-MRGPRF-6:1 knockout stable macrophage. The sgRNA targeting lnc-MRGPRF-6:1 was designed using CRISPOR (http://crispor.tefor.net) [17]. Two targets were designed for each location, and two locations were designed for lnc-MRGPRF-6:1. Lentivirus encoding Cas9 nuclease and sgRNA targeting lnc-MRGPRF-6:1 or sgRNA control were constructed and packed by GENECHEM (Shanghai, China). Macrophages were infected by lentivirus for 24 h, and lnc-MRGPRF-6:1 knockout stable cells were sieved with a full medium containing puromycin for 72 h.

### 2.8. Activate TLR4-MyD88-MAPK Signaling Pathways

lnc-MRGPRF-6:1 knockout macrophage (KO) and control cells (1 × 10^6^/well) were inoculated in 6-well plates with 2 mL per well and incubated with 100 ng/mL LPS for 24 h.

### 2.9. RNA Extraction and Quantitative Real-Time PCR

Total RNA was extracted from the cells using the RNeasy Plus Mini Kit (QIAGEN, 74134) , from the plasma using BIOG cfDNA Easy Kit (BIOG, 51028) and from exosomes by RNA Purification Kit (EZBioscience, EZB-EXO-RN1). Total RNA was reverse transcribed into cDNA by using PrimeScript™ RT Reagent Kit (Takara, RR037A). RT-qPCR was conducted using SYBR® Premix Ex Taq™ II (Takara, RR420A) on StepOnePlus Real-Time PCR System (ABI, USA). U6 was used as an internal reference. The primers of all genes were synthesized by GenScript (Nanjing, China). Primer sequences are listed in Table 1. Equation 2^−∆∆CT^ is used to analyze the relative expression data.

### 2.10. Cytokine Measurement via Enzyme-Linked Immunosorbent Assay (ELISA)

The cell culture supernatant of each treatment group was collected, and the secretion of TNF-*α* (Beyotime, PI660), TNF-*β* (Beyotime, PT903), and IL-10 (Beyotime, PI528) in the cell culture medium was detected according to the protocols.

### 2.11. Flow Cytometry

Annexin-V-FITC apoptosis kit (KeyGEN BioTECH, KGA108) was utilized for apoptosis determination. 10^6^ cells were dispersed with EDTA-free trypsin, washed with PBS, and resuspended in 500 *μ*L binding buffer and were then stained with FITC-labeled annexin V and PI. Apoptotic cell rate (%) = annexin + /PI − cells (%) + annexin + /PI + cells (%). Flow cytometry was used to detect the fluorescence intensity of foam cell samples with ROS Assay Kit (Beyotime, S0033S), and average fluorescence intensity (MFI) represented ROS content.

### 2.12. Oil Red O Staining Test

THP-1 cells were induced to differentiate into foam cells. Dye with filtered solution of Oil Red O (Solarbio, G1262) was used for lipid staining in foam cells. The cells were finally observed and photographed microscopically.

### 2.13. Cell Counting Kit-8 (CCK-8) Assay

A CCK-8 assay tested the proliferation of cells (Dojindo, CK04). After 48 h of incubation, the supernatant was refreshed with RPMI-1640 medium containing CCK-8 for another 2 h. An automated microplate reader was used to read the optical density (OD) at 450 nm.

### 2.14. Western Blot

Protein samples were obtained by cleavage of cells using RIPA lysis buffer (Beyotime, P0013B). After being measured with bicinchoninic acid kit (Beyotime, P0010S), normalized volumes of protein samples were isolated on SDS-PAGE on a 10% gel and transferred onto polyvinylidene fluoride membranes. The transfer membrane was then washed in a wash buffer and sealed in 5% BSA TBST for 1 h, followed by overnight incubation with primary antibody at 4°C. Then, HRP-labeled goat anti-rabbit IgG was stained for 1 h. After the developer solution is added, the film is viewed under Bio-Rad chemiluminescence imaging system. The primary antibodies used in experiments were as follows: P38 (CST, 9252T), p-P38 (CST, 4511T), TLR4 (NOVUS, NB-100-56580), MyD88 (FineTest, FNab10314), GAPDH (Beyotime, AF1186), JNK (CST, 9252T), p-JNK (CST, 4668T), ERK (CST, 4695T), p-ERK (CST, 4370T), and Tubulin (Beyotime, AF1216).

### 2.15. RNA Sequencing and Data Analysis

Total RNA was extracted from the cells using Trizol (Invitrogen, 15596018). RNA-seq was performed by BGISEQ-500 platform of Beijing Genomics Institute (BGI). Data analyses were carried out with BGI Online platform (http://www.bgionline.cn).

### 2.16. Statistical Analysis

GraphPad Prism 7 (GraphPad Software, USA) software and WPS Office 3 (Kingsoft Software, China) were used for statistical analysis. Quantitative data between the two groups were evaluated using Student's *t*-test and Mann–Whitney *U* tests. Spearman correlation analysis confirmed the correlation. *P* < 0.05 was considered statistically significant.

## 3. Results

### 3.1. lnc-MRGPRF-6:1 Is Highly Expressed in the Plasma Exosomes of CAD Patients and Is Positively Correlated with the Expression of Inflammatory Cytokines in Plasma

**Figure 1:**
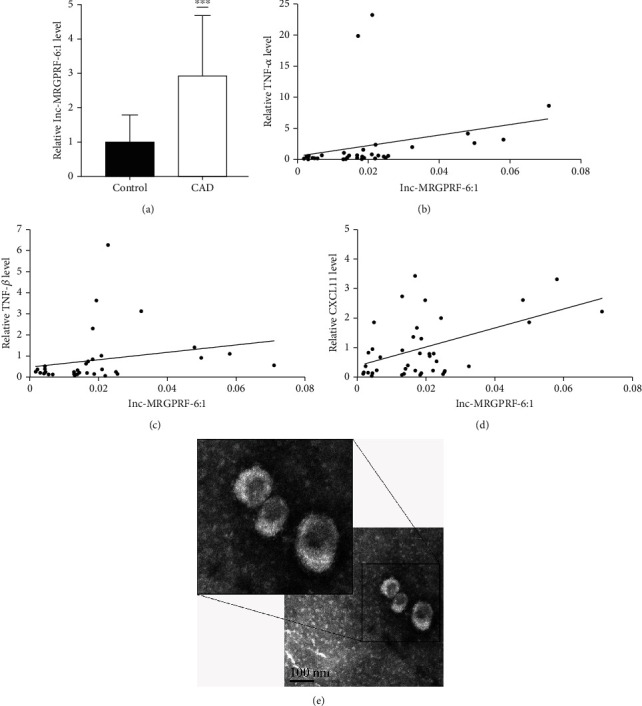
lnc-MRGPRF-6:1 significantly increased in plasma exosomes of CAD patients and was associated with inflammatory factors in plasma. (a) The mRNA level of lnc-MRGPRF-6:1 in the plasma exosomes was detected by RT-qPCR. (b–d) Scatter plot of Spearman correlation analysis between lnc-MRGPRF-6:1 and TNF-*α* (Spearman *r* = 0.544, *P* = 0.001), TNF-*β* (Spearman *r* = 0.469, *P* = 0.005), and CXCL11 (Spearman *r* = 0.376, *P* = 0.018) expression levels in plasma. (e) Electron microscopy image of exosome enrichment isolated from plasma (scale bar, 100 nm). CAD vs. control, ^∗∗∗^*P* < 0.001. CAD: coronary atherosclerotic disease.

We successfully isolated exosomes and characterized the vesicles by TEM. TEM shows the enriched fraction of exosome-like vesicles (Figure 1(e)). A total of 40 subjects (20 CAD patients and 20 healthy controls) were selected for the study. It was shown that the lnc-MRGPRF-6:1 levels in the plasma exosomes of CAD patients were significantly higher than those of the control group (Figure 1(a)). Compared with the control group, as shown in Table 2, mRNA levels of TNF-*α*, TNF-*β*, and CXCL11 in the plasma of CAD patients were significantly increased with U6 as an internal reference. Further research demonstrated that lnc-MRGPRF-6:1 was positively correlated with TNF-*α* (Spearman *r* = 0.544, *P* = 0.001), TNF-*β* (Spearman *r* = 0.469, *P* = 0.005), and CXCL11 (Spearman *r* = 0.376, *P* = 0.018) in plasma (Figures 1(b)–1(d)).

### 3.2. lnc-MRGPRF-6:1 Knockout Blocks ox-LDL-Induced Macrophage Foam Cell Formation

**Figure 2:**
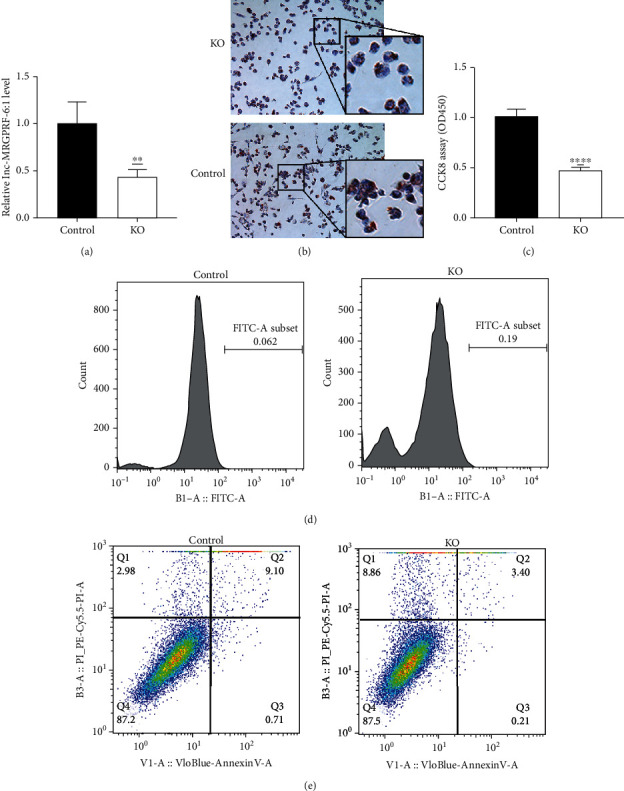
Knockout of lnc-MRGPRF-6:1 can reduce the lipid accumulation and ROS levels of foam cells, significantly increase the viability of macrophages, and inhibit cell apoptosis. After the lnc-MRGPRF-6:1 knockout macrophage was induced to differentiate with the control cells, the cells were stimulated with 50 mg/mL ox-LDL for 24 h. (a) The expression level of lnc-MRGPRF-6:1 between KO and control was detected by RT-qPCR. (b) Image of Oil Red O staining. (c) Viability of macrophage. (d) ROS levels in macrophage. (e) Apoptosis level of macrophage. KO vs. control, ^∗^*P* < 0.05, ^∗∗^*P* < 0.01, ^∗∗∗^*P* < 0.001, and ^∗∗∗∗^*P* < 0.0001. KO: lnc-MRGPRF-6:1 knockout macrophage; control: macrophage infected by control lentivirus.

As mentioned above, THP-1 macrophage cells were used to establish lnc-MRGPRF-6:1 knockout macrophage model via the CRISPR/Cas9 system. It was confirmed that the expression of lnc-MRGPRF-6:1 was downregulated significantly in knockout macrophage (KO) compared with macrophage infected by control lentivirus (control) (Figure 2(a)). Subsequently, THP-1 macrophage foam cell model was established by using ox-LDL. Oil Red O staining analysis indicated lipid accumulation in ox-LDL-induced macrophage foam cells was reduced after lnc-MRGPRF-6:1 knockout (Figure 2(b)). Moreover, ROS generation was dramatically decreased in lnc-MRGPRF-6:1 knockout macrophage foam cells (Figure 2(d)). In addition, CCK-8 assay and flow cytometry suggested that knockdown of lnc-MRGPRF-6:1 decreased cell viability and inhibited cell apoptosis (Figures 2(c) and 2(e)).

### 3.3. lnc-MRGPRF-6:1 Is Significantly Upregulated in M1 Macrophage

**Figure 3:**
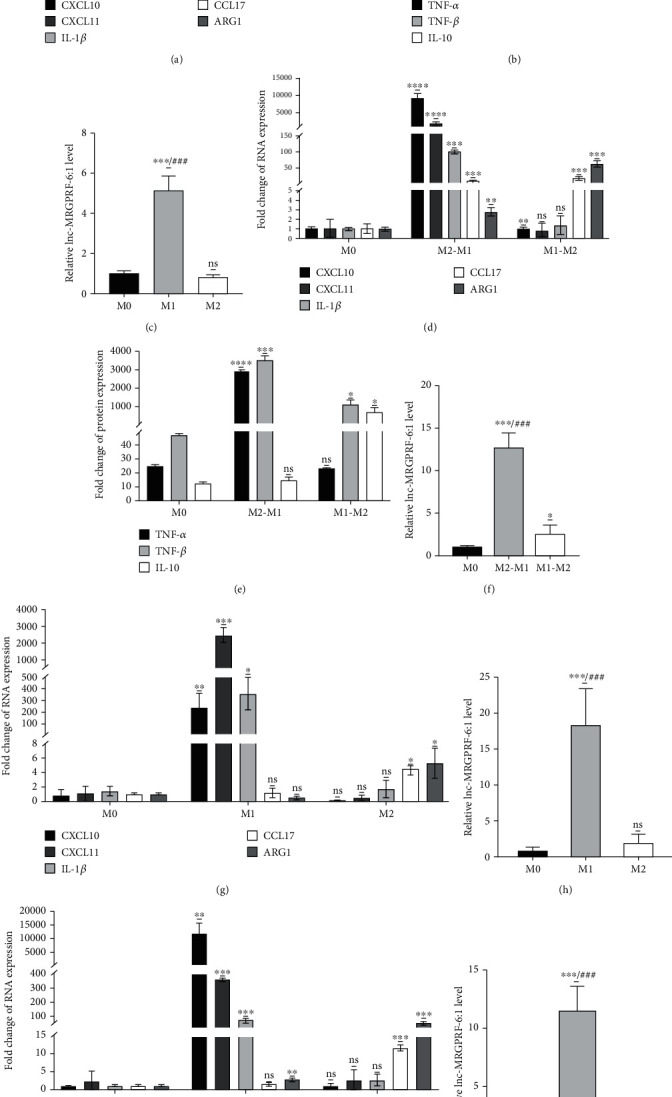
lnc-MRGPRF-6:1 was highly expressed in M1 macrophage. (a, b, g) M1 polarization was stimulated by IFN-*γ*/LPS, and M2 polarization was stimulated by IL-4. The levels of TNF-*α*, TNF-*β*, and IL-10 in the supernatant were detected by ELISA, and the mRNA levels of CXCL10, CXCL11, IL-1*β*, ARG1, and CCL17 were detected by RT-qPCR. (c, h) The expression levels of lnc-MRGPRF-6:1 in macrophages with different phenotypes were detected by RT-qPCR. (d, e, i) After repolarization, CXCL10, CXCL11, IL-1*β*, ARG1, and CCL17 levels were detected by RT-qPCR, and TNF-*α*, TNF-*β*, and IL-10 were detected by ELISA. (f, j) The expression levels of lnc-MRGPRF-6:1 in repolarization macrophages were detected by RT-qPCR. (a–c, g, h) M1/M2 vs. M0, ns: not statistically significant; ^∗^*P* < 0.05, ^∗∗^*P* < 0.01, ^∗∗∗^*P* < 0.001, and ^∗∗∗∗^*P* < 0.0001; M1 vs. M2, ^###^*P* < 0.001; (d–f, i, j) M2-M1/M1-M2 vs. M0, ns: not statistically significant; ^∗^*P* < 0.05, ^∗∗^*P* < 0.01, ^∗∗∗^*P* < 0.001, and ^∗∗∗∗^*P* < 0.0001; M2-M1 vs. M1-M2, ^##^*P* < 0.01.

After being induced by IFN-*γ* and LPS, both the levels of CXCL10, CXCL11, IL-1*β*, TNF-*α*, and TNF-*β* protein secretions were significantly increased in THP-1 macrophage, which suggested that THP-1 macrophage was polarized to M1 type (M1) successfully (Figures 3(a) and 3(b)). Concurrently, being treated with IL-4, both the levels of CCL17, ARG1, and IL-10 protein secretion were dramatically increased in THP-1 macrophage, which suggested that THP-1 macrophage was polarized to M2 type (M2) successfully (Figures 3(a) and 3(b)). It is worth noting that, compared with untreated THP-1 macrophage (M0), the expression of lnc-MRGPRF-6:1 in M1 was increased notably while the expression in M2 has no marked change (Figure 3(c)).

For further identification, we intend to analyze the expression of lnc-MRGPRF-6:1 in macrophage repolarization models. It was observed that the addition of IFN-*γ* and LPS in M2 (M2-M1) culture medium could increase the levels of M1-related cytokines CXCL10, CXCL11, IL-1*β*, TNF-*α*, and TNF-*β* and decrease the levels of M2-related cytokines CCL17, ARG1, and IL-10 (Figures 3(d) and 3(e)). Similarly, the addition of IL-4 in M1 (M1-M2) culture medium increased the levels of M2-related cytokines in macrophage, but decreased the levels of M1-related cytokines (Figures 3(d) and 3(e)). It was confirmed that macrophage repolarization models were constructed successfully. Unsurprisingly, lnc-MRGPRF-6:1 expression in M2-M1 was increased apparently (Figure 3(f)).

To further investigate the high expression of lnc-MRGPRF-6:1 in M1 macrophage, we induced human monocyte-derived macrophage to polarize M1 type using IFN-*γ* and LPS; lnc-MRGPRF-6:1 was still significantly elevated (Figures 3(g) and 3(h)). Moreover, the expression of lnc-MRGPRF-6:1 in M2-M1 was also increased in human monocyte-derived macrophage repolarization model (Figures 3(i) and 3(j)).

These facts suggest that the expression of lnc-MRGPRF-6:1 was upregulated in M1 macrophage, and the expression will change with the phenotype transformation of the macrophage.

### 3.4. lnc-MRGPRF-6:1 Knockout Inhibits the Polarization of M1 Macrophage

**Figure 4:**
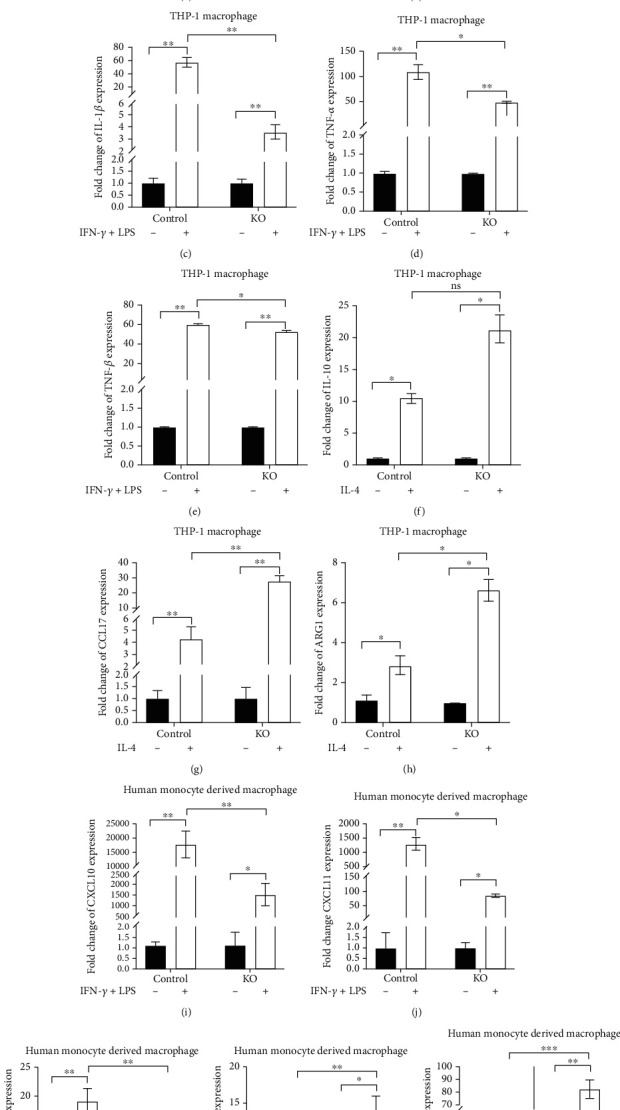
Knockout of lnc-MRGPRF-6:1 inhibits the formation of M1 macrophage and reduces the secretion of inflammatory cytokines. (a–c, i–k) The mRNA level of CXCL10, CXCL11, and IL-1*β* in M1-type macrophage was detected by RT-qPCR. (g, h, l, m) The expression of CCL17 and ARG1 in M2-type macrophage. (d, e) The protein levels of TNF-*α* and TNF-*β* in M1-type macrophage were detected by ELISA. (f) The expression of IL-10 in M2-type macrophage. ns: not statistically significant; ^∗^*P* < 0.05, ^∗∗^*P* < 0.01, ^∗∗∗^*P* < 0.001, and ^∗∗∗∗^*P* < 0.0001.

After being induced by IFN-*γ* and LPS, the levels of CXCL10, CXCL11, and IL-1*β* in both lnc-MRGPRF-6:1 knockout macrophage (KO) and control were significantly increased (Figures 4(a)–4(c)). Furthermore, the increases in KO were inferior to control. In the meantime, compared with control, TNF-*α* and TNF-*β* secretions were increased apparently in both KO and control, and the increasing levels in KO were also less than those in control (Figures 4(d) and 4(e)).

Meanwhile, induced by IL-4, the CCL17 and ARG1 levels in both KO and control were dramatically increased, and the increases in KO were great than those in the control group (Figures 4(g) and 4(h)). Similarly, the IL-10 secretions in both KO and control were also markedly increased (Figure 4(f)). Interestingly, the difference of IL-10 secretion level between KO and control was not statistically significant.

To further clarify whether lnc-MRGPRF-6:1 knockout inhibited M1 macrophage polarization, we infected human monocyte with lentivirus to knock out lnc-MRGPRF-6:1. After being induced into M1 and M2 macrophage, the increase of M1 markers (CXCL10, CXCL11, and IL-1*β*) after lnc-MRGPRF-6:1 knockout was significantly lower than that of the control group (Figures 4(i)–4(k)), while the increase of M2 markers (CCL17, ARG1) was higher (Figures 4(l) and 4(m)).

These results suggest that lnc-MRGPRF-6:1 knockout effectively inhibits the polarization of M1 macrophage.

### 3.5. lnc-MRGPRF-6:1 May Regulate Macrophage Polarization via the TLR4-MyD88-MAPK Pathway

**Figure 5:**
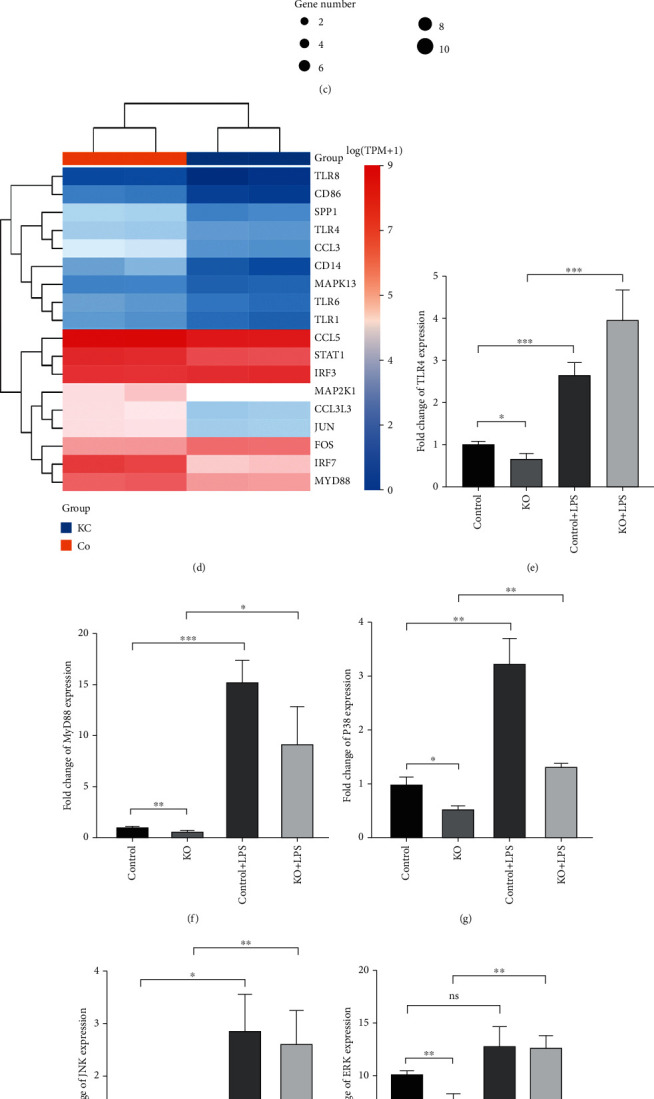
lnc-MRGPRF-6:1 may regulate macrophage polarization via the TLR4-MyD88-MAPK pathway. RNA sequencing was performed between lnc-MRGPRF-6:1 knockout macrophage (KO) and macrophage infected by control lentivirus (control). (a) Volcano plot shows differentially expressed genes between KO and control. (b, c) GO and KEGG analysis according to RNA sequencing data. (d) Hierarchical cluster analysis of differentially expressed mRNAs in KO compared to control (partially shown); (e–l) mRNA levels of TLR4, MyD88, P38, JNK, ERK, CXCL10, CXCL11, and IL-1*β* between KO, control, and after LPS induction were detected by RT-qPCR. (m) Expression of TLR4, MyD88, JNK, ERK, P38, p-P38, p-JNK, and p-ERK between KO and control were analyzed by Western blotting. KO: lnc-MRGPRF-6:1 knockout macrophage; control: macrophage infected by control lentivirus.

RNA sequencing was performed between lnc-MRGPRF-6:1 knockout macrophage (KO) and macrophage infected by control lentivirus (control). Transcriptome analysis showed that the expression levels of 32 genes were up-regulated and 191 down-regulated in THP-1 macrophage after lnc-MRGPRF-6:1 knockout with ∣Log_2_FC | >1 and *P* < 0.05 (Figure 5(a)). Gene Ontology (GO) and KEGG pathway enrichment analyses were then carried out for further analysis.

As shown in Figure 5(b), functions and processes associated with immune were enriched after lnc-MRGPRF-6:1 knockout, including *immune system processes*, *innate immune response*, and *inflammatory response*. In innate immunity, macrophages produce proinflammatory mediators by activating several receptors that recognize pathogens, including the toll-like receptor family (TLRs) [18]. Coincidentally, the toll-like receptor signaling pathway was enriched by KEGG pathway analysis (Figure 5(c)). After LPS stimulation, activated TLR4 can trigger the downstream MAPK signaling pathway through the MyD88-dependent pathway [19]. Fascinatingly, according to RNA sequencing data, expressions of TLR4, MyD88, and P38 were significantly changed after lnc-MRGPRF-6:1 knockout (Figure 5(d)).

Considering MAPK including JNK, ERK, and P38 signal transduction pathway, we still verified JNK and ERK pathways, although RNA-seq showed no significant changes in JNK and ERK expression in MAPK after lnc-MRGPRF-6:1 knockout.

Subsequently, we verified the expression of the genes above-mentioned by RT-PCR and Western blotting. In Figures 5(e)–5(i) and 5(m), TLR4, MyD88, P38, phosphorylation of P38, JNK, and phosphorylation of JNK expressions were decreased in both transcription and protein level after lnc-MRGPRF-6:1 knockout. Although ERK was decreased at the transcription level, there was no significant difference in the protein level, which suggested that lnc-MRGPRF-6:1 might regulate macrophage polarization through the TLR4-MyD88-MAPK signaling pathway.

To further investigate that lnc-MRGPRF-6:1 regulates macrophage polarization through the TLR4-MyD88-MAPK signaling pathway, we used LPS, the activator of TLR4, to activate this signaling pathway. LPS induced intense activation of TLR4, MyD88, P38, phosphorylation of P38, JNK, and phosphorylation of JNK in the KO group (Figures 5(e)–5(i) and 5(m)); meanwhile, M1 markers (CXCL10, CXCL11, and IL-1*β*) were significantly elevated in the KO group after LPS induction (Figures 5(g)–5(l)).

## 4. Discussion

AS, as a chronic inflammatory vascular disease, is the pathological basis of CAD, which is a serious threat to human health. It is demonstrated that lncRNAs play important regulatory roles in inflammatory diseases, including AS [20]. In a preliminary work, we identified that lnc-MRGPRF-6:1 was significantly upregulated in the plasma of CAD patients. We want to explore whether there is a relationship between lnc-MRGPRF-6:1 and AS.

Foam cells derived from macrophages are associated with the initial stage of AS [21]. Therefore, we investigated the effect of lnc-MRGPRF-6:1 expression on macrophage foam cell formation. We first established lnc-MRGPRF-6:1 knockout macrophage model and then analyzed the lipid accumulation, ROS generation, viability, and apoptosis of model cells. The results show that lnc-MRGPRF-6:1 knockout blocks ox-LDL-induced macrophage foam cell formation, and lnc-MRGPRF-6:1 promotes the production of ROS, apoptosis, and viability loss of model cells. Foam cells derived from macrophages are the main cell type of early AS lesions [22]. Macrophages are Foam cells derived from macrophages are the main cell type of early AS lesions [22]. Macrophages are induced into foam cells after uptake of ox-LDL, which constitutes one of the important steps in the development of AS plaques [23]. In the present study, lnc-MRGPRF-6:1 promotes the production of ROS, which oxidizes LDL to form ox-LDL. Increased apoptosis and viability of macrophages can lead to lipid aggregation and further inflammation and promote the development of AS plaques [23]. These results suggest that lnc-MRGPRF-6:1 may play a crucial role in the development of AS.

Previous studies demonstrate that macrophage-mediated inflammation plays an essential role in the development of AS. After overtaking ox-LDL, macrophage transforms into foam cell under the intima of the coronary artery and recruits many proinflammatory factors to initiate the disease [24]. Afterwards, various inflammatory factors are released and lead to accelerating AS.

Interestingly, in our study, further research demonstrated lnc-MRGPRF-6:1 was positively correlated with TNF-*α*, TNF-*β*, and CXCL11 in CAD patients' plasma, which indicates that lnc-MRGPRF-6:1 may participate in the regulation of macrophage-mediated inflammation in AS.

Polarization is deeply involved in the regulation of macrophage-mediated inflammation [25, 26]. Macrophages could be polarized into classical activated M1 type and replacement activated M2 type. The M1 phenotype macrophage secretes inflammatory cytokines, which leads to recruiting inflammatory cells and aggravates inflammation and plaque formation [27]. In contrast, M2 phenotype macrophage, as an anti-inflammatory cell, could block the recruitment of inflammatory cells, inhibit the release of inflammatory cytokines, and reduce the formation of foam cells [28]. Therefore, the disbalance of macrophage polarization is closed related to the occurrence and development of AS.

Initially, we analyze the expression of lnc-MRGPRF-6:1 in both M1 and M2 phenotype macrophages. For further research, we analyze the expression of lnc-MRGPRF-6:1 in the repolarization models of M1 and M2 macrophages once again. lnc-MRGPRF-6:1 is significantly upregulated in M1 macrophage. It is worth noting that the unchanged thing is the expression of lnc-MRGPRF-6:1 which was upregulated in M1 macrophage. Moreover, we observe that lnc-MRGPRF-6:1 knockout inhibits the polarization of M1 macrophage. These facts suggest that lnc-MRGPRF-6:1 may modulate the macrophage-mediated inflammation through transforming its polarization.

However, the problem is how lnc-MRGPRF-6:1 regulates macrophage polarization. We conducted transcriptome sequencing between lnc-MRGPRF-6:1 knockout macrophage and control macrophage. Subsequently, GO enrichment analysis indicates that functions and processes associated with immune were enriched after lnc-MRGPRF-6:1 knockout. Coincidentally, the toll-like receptor signaling pathway, which is closely related to immune, was enriched by KEGG pathway analysis.

TLRs, regulating immune cell survival and proliferation, are involved in the first line of defense against invading pathogens in organisms and play important roles in inflammation [29]. TLRs play a central role in macrophage activation, differentiation, and polarization. In TLRs, TLR4 is a pattern recognition receptor in response to LPS. The binding of LPS to TLR4 induces phosphorylation of IRAK1, PI3K, or MAPKs, which are involved in host defense mechanisms by recognizing pathogens or acting as receptors for proinflammatory cytokines. The TLR-induced inflammatory response triggers the downstream MAPK signaling pathway through the MyD88 pathway [30]. MAPK consists of a group of serine/threonine protein kinases that mediate signaling from the cell surface to the nucleus following activation by various extracellular stimuli. The MAPK signaling pathway has been found in mammalian cells including JNK, ERK, and P38 pathways [19], and it can be induced by LPS-activated TLR4 [31].

Fascinatingly, expressions of TLR4, MyD88, and P38 were significantly changed after lnc-MRGPRF-6:1 knockout according to RNA sequencing data. Further analyses demonstrate that the expression levels of TLR4, MyD88, p-P38, and p-JNK were significantly decreased after knockout. After LPS induced lnc-MRGPRF-6:1 knockout macrophage model, the TLR4-MyD88-MAPK pathway was activated; meanwhile, CXCL10, CXCL11, and IL-1*β*, markers of M1, were also significantly increased.

Based on these findings, we speculate that lnc-MRGPRF-6:1 promotes macrophage-mediated inflammation through the TLR4-MyD88-MAPK signaling pathway which regulates macrophage M1 polarization.

In summary, the present study clarifies that lnc-MRGPRF-6:1 promotes macrophage-mediated inflammation by regulating macrophage M1 polarization, which may play a crucial role in the development of AS. Keeping the balance of macrophage polarization for reducing foam cell formation and alleviating inflammation has become the ideas for AS treatment strategies, and lnc-MRGPRF-6:1 might become a potential therapeutic target for AS.

## Figures and Tables

**Table 1:** The primer sequences in the present study.

Gene	Primer sequences (5′-3′)
TNF-*α* forward	AGGACACCATGAGCACTGAA
TNF-*α* reverse	CCGATCACTCCAAAGTGCAG
TNF-*β* forward	CTTCGTGCTTTGGACTACCG
TNF-*β* reverse	AGACGTTCAGGTGGTGTCAT
CXCL11 forward	CCTTGGCTGTGATATTGTGTGCTA
CXCL11 reverse	CCTATGCAAAGACAGCGTCCTC
CXCL10 forward	GGCCATCAAGAATTTACTGAAAGCA
CXCL10 reverse	TCTGTGTGGTCCATCCTTGGAA
CCL17 forward	CTTCTCTGCAGCACATCCAC
CCL17 reverse	TGGTACCACGTCTTCAGCTT
U6 forward	AACGCTTCACGAATTTGCGT
U6 reverse	CTCGCTTCGGCAGCACA
lnc-MRGPRF-6:1 forward	AGGGACAGGAAGATGGTTGGC
lnc-MRGPRF-6:1 reverse	GATGAGCAGAATGGTCGTGAGG
IL-1*β* forward	CTCTCTCCTTTCAGGGCCAA
IL-1*β* reverse	GCGGTTGCTCATCAGAATGT
ARG1 forward	GAAAGGCTGGTCTGCTTGAG
ARG1 reverse	CAGCACCAGGCTGATTCTTC
ERK forward	TTTCCTCTGGATCAGCGTGT
ERK reverse	GAGGCCTGTGAGCATTTCTG
JNK forward	GTTCCTGACGTGCACTCTTC
JNK reverse	GGATGCTTCTTTGCACACCA
TLR4 forward	GCCACATGTCAGGCCTTATG
TLR4 reverse	TTGGTTGAAATGCCCACCTG
MyD88 forward	CAGCTCTGAGCCATTCACAC
MyD88 reverse	CCAGCATGTAGTCCAGCAAC
P38 forward	AATCCTCACCATCCACAGCA
P38 reverse	GCTTAGAGTCCAGGCTTCCA

**Table 2:** Relative expressions of TNF-*α*, TNF-*β*, and CXCL11 at the transcription level between CAD patients and controls (U6 as an internal reference). The data were expressed as median (1st/3rd quartiles).

	TNF-*α*	TNF-*β*	CXCL11
Control (*n* = 20)	0.22 (0.14, 0.35)	0.23 (0.12, 0.27)	0.21 (0.11, 0.76)
CAD (*n* = 20)	1.52 (0.46, 3.85)	0.87 (0.43, 1.40)	1.40 (0.49, 2.62)
*P*	<0.001	<0.001	0.001

## Data Availability

The data used to support the findings of this study are included within the article.
